# A Comparative Analysis of Floral Scent Compounds in Intraspecific Cultivars of *Prunus mume* with Different Corolla Colours

**DOI:** 10.3390/molecules25010145

**Published:** 2019-12-30

**Authors:** Tengxun Zhang, Fei Bao, Yongjuan Yang, Ling Hu, Anqi Ding, Aiqin Ding, Jia Wang, Tangren Cheng, Qixiang Zhang

**Affiliations:** Beijing Advanced Innovation Center for Tree Breeding by Molecular Design, Beijing Key Laboratory of Ornamental Plants Germplasm Innovation & Molecular Breeding; National Engineering Research Center for Floriculture; Beijing Laboratory of Urban and Rural Ecological Environment; Key Laboratory of Genetics and Breeding in Forest Trees and Ornamental Plants of Ministry of Education; School of Landscape Architecture, Beijing Forestry University, Beijing 100083, China; zhangtengxun@163.com (T.Z.); baofei@bjfu.edu.cn (F.B.); yongjuanyang_bjfu@163.com (Y.Y.); Gloria_hu95@outlook.com (L.H.); angeldaq@126.com (A.D.); 18363973580@163.com (A.D.); wangjia8248@163.com (J.W.); chengtangren@163.com (T.C.)

**Keywords:** floral scent, *Prunus mume*, intraspecific cultivars, volatile compounds, phenylpropanoids/benzenoids, HS-SPME-GC-MS

## Abstract

*Prunus mume* is the only fragrant flowering species of *Prunus*. According to the previous studies, benzyl acetate and eugenol dominate its floral scent. However, the diversity of its floral scents remains to be elucidated. In this work, the floral volatiles emitted from eight intraspecific cultivars of *P. mume* with white, pink and red flowers, were collected and analyzed using headspace solid-phase microextraction combined with gas chromatograms-mass spectrometry (HS-SPME-GC-MS). In total, 31 volatile compounds were identified, in which phenylpropanoids/benzenoids accounted for over 95% of the total emission amounts. Surprisingly, except for benzyl acetate and eugenol, several novel components, such as benzyl alcohol, cinnamyl acohol, cinnamy acetate, and benzyl benzoate were found in some cultivars. The composition of floral volatiles in cultivars with white flowers was similar, in which benzyl acetate was dominant, while within pink flowers, there were differences of floral volatile compositions. Principal component analysis (PCA) showed that the emissions of benzyl alcohol, cinnamyl alcohol, benzyl acetate, eugenol, cinnamyl acetate, and benzyl benzoate could make these intraspecific cultivars distinguishable from each other. Further, hierarchical cluster analysis indicated that cultivars with similar a category and amount of floral compounds were grouped together. Our findings lay a theoretical basis for fragrant plant breeding in *P. mume*.

## 1. Introduction

For ornamental plants, floral fragrance is an attractive and significant character, playing a crucial role in plant–animal communication, including attracting pollinators and defense against pathogens [1]. Also, floral scent can increase aesthetic value and attract visitors [2]. The trait of floral scent is complex and composed of a series of low molecular weight volatile organic compounds (VOCs) [3]. The composition and number of VOCs vary among different species or even in interspecific and intraspecific cultivars [4,5]. In recent years, more and more studies focusing on floral fragrance profiles have been performed in many fragrant plants, including *Lilium* [6,7], *Gelsemium sempervirens* [8], *Chimonanthus praecox* [9], *Camellia* [4], *Polianthes tuberosa* [5], and bearded irises [10], etc. VOCs released from these flowers are basically divided into four classes by the biosynthesis origin: terpenoids, phenylpropanoids/benzenoids, fatty acid derivatives, and compounds containing nitrogen/sulfur [11].

*P. mume* (mei) from Rosaceae, is a famous ornamental and fruit tree blooming in early spring within January and February, and it is mainly cultivated in Yangtze valley that locates in the subtropical monsoon climate zone [12]. It emits recognizable and pleasant scents, making it distinguishable from other species in *Prunus*, such as *P. sibirica*, *P. armeniaca,* and *P. persica* [13]. It has been reported that the aromatic benzenoid, benzyl acetate, is the main component in its floral volatiles [14]. However, very little is known about the diversity of the floral scents in its cultivars with different coloring corollas, which includes two groups: one in white and light yellow without anthocyanin, the other one in light pink, pink and fuchsia with anthocyanin [15]. As reported, there is a close connection between metabolic biosynthesis of floral volatiles and flower coloration, because aromatic benzenoids and anthocyanin are derived from their common precursor, aromatic amino acid phenylalanine, through phenylpropanoids biosynthesis pathway [16]. There are some researches focusing on the relation between floral scent and flower colour, which usually mentioned the regulation of transcription factors (TF). A TF from MYB family, production of anthocyanin pigment 1 (*pap1*) in *Arabidopsis thaliana* can activate phenylpropanoids pathway to enhance the emission of phenylpropanoid/benzenoid compounds and accumulate anthocyanin pigments [17,18]. In Petunia hybrids, flower pigment and scent emission are also co-regulated by *PH4* [16]. Whether the floral fragrance of *P. mume* intraspecific cultivars with different flower colours varies or not awaits further investigation. *P. mume* has been recognized to be an essential material to investigate the floral metabolic characteristics.

The present study aims to explore the diversity of floral scents in *P. mume* intraspecific cultivars with different corolla colours. It reports the floral scents through characterizing the emitted VOCs with HS-SPME-GC-MS. First, the specific floral volatile compounds from the investigated cultivars were identified. We analyzed the emission composition referring to their percentages and absolute amounts. Then, PCA was performed to categorize the intraspecific cultivars and clarify the major contributing component. Further, the eight cultivars were classified into two groups in terms of the absolute amount of main floral components through hierarchical cluster analysis. This study will provide a better understanding of the floral scent chemistry in *P. mume* cultivars and help breeders to develop new fragrant cultivars.

## 2. Results

### 2.1. Floral Volatiles in the Eight P. mume Cultivars

**Figure 1:**
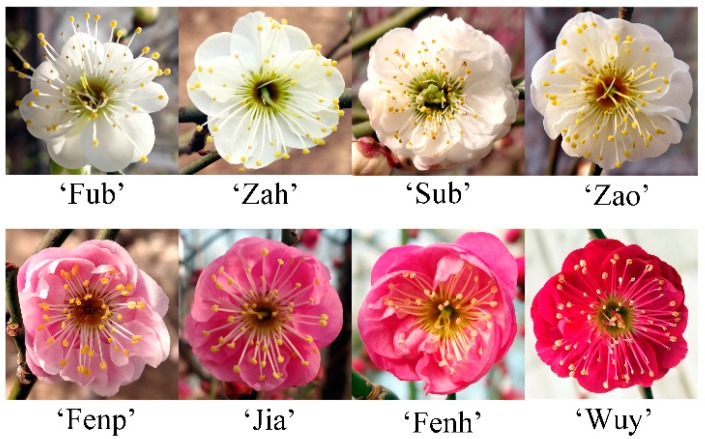
The flowers of eight intraspecific cultivars with different colours.

A total of 31 different floral volatiles were identified in the eight investigated cultivars with different corolla colours (Figure 1). The colour parameter and number of detected compounds in different cultivars were summarized in Table 1.

**Figure 2:**
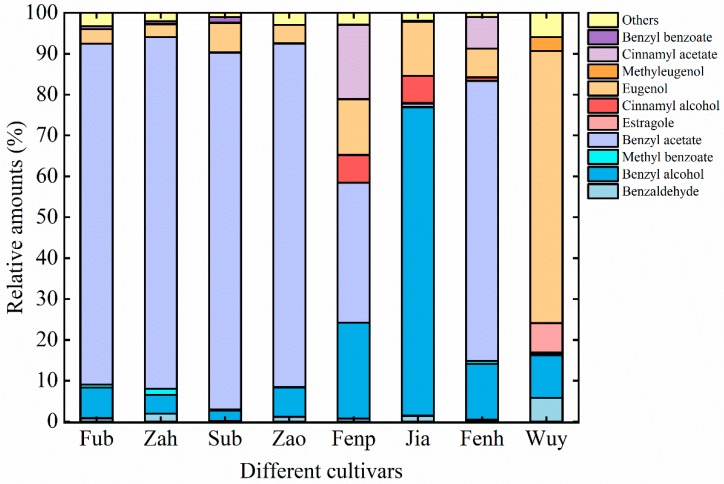
Major components (>1%) of floral volatiles emitted from *P. mume* cultivars.

The composition of VOCs varied obviously among these cultivars. VOCs were classified according to their chemical categories, and their relative amounts are listed in Supplementary Table S1. The detected floral VOCs included 17 phenylpropanoids/benzenoids, 10 fatty acid derivatives, 4 terpenoids. Phenylpropanoids/benzenoids, as the most prior VOCs, accounted for as high as above 95% of total emissions. Ten compounds represented more than 1% of total emission in any cultivar (Figure 2), among which benzyl acetate, benzyl alcohol, eugenol, cinnamyl acetate, and cinnamyl alcohol accounted for a large percentage of total amounts. Except for in ‘Jia’ and ‘Wuy’, benzyl acetate accounted for the highest percentage in the other six cultivars and varied from 34.23% to 87.26%. Benzyl alcohol was emitted from all cultivars, while its relative amount ranged from 2.53% to 75.43%. Similarly, eugenol was detected in all samples, and its relative abundance reached as the highest as 70.92% in ‘Wuy’. Cinnamyl acetate and cinnamyl alcohol only presented in three of them. Their relative amounts ranged from 0.08% to 18.14% and from 0.88% to 6.81%, respectively.

The fragrance was suggested to be related to the absolute number of compounds, which was not always consistent with their relative amounts for the different adsorption coefficient of VOCs. As shown in Table 2, except for ‘Fenp’, the emission amount of benzyl acetate was also the most in five of the six cultivars mentioned above, which ranged from 239.2 to 1333.0 μg·g^−1^ fresh weight·h^−1^, indicating that benzyl acetate may play a major role in their floral fragrance. Flowers of ‘Fenp’ emitted the highest amount of cinnamyl acetate at 810.3 μg·g^−1^ fresh weight·h^−1^, which was much larger than other chemicals. It indicated cinnamyl acetate contributed the most to floral fragrance of ‘Fenp’. The emission of benzyl alcohol varied from 38.2 μg·g^−1^ fresh weight·h^−1^ in ‘Sub’ to 1094.0 μg·g^−1^ fresh weight·h^−1^ in ‘Jia’, whose fragrance might be dominated by benzyl alcohol. In addition, eugenol was emitted differently from 36.39 to 406.33 μg·g^−1^ fresh weight·h^−1^. Though accounting for the largest proportion of total floral emission amounts, the concentration of eugenol in ‘Wuy’ was lower than in ‘Zao’ while approximated it in ‘Jia’. Cinnamyl alcohol emission amounts were close between in ‘Fenp’ and ‘Jia’, which were more than that in ‘Fenh’. Benzyl benzoate was only emitted from ‘Fub’, ‘Zah’ and ‘Sub’, which amounts changed from 95.00 to 157.47 μg·g^−1^ fresh weight·h^−1^. In total, these results suggested that benzyl acetate, benzyl alcohol, cinnamyl acetate, and eugenol were the main constituents of *P. mume* floral scents.

### 2.2. Principal Component Analysis of Floral Scent Emission

**Figure 3:**
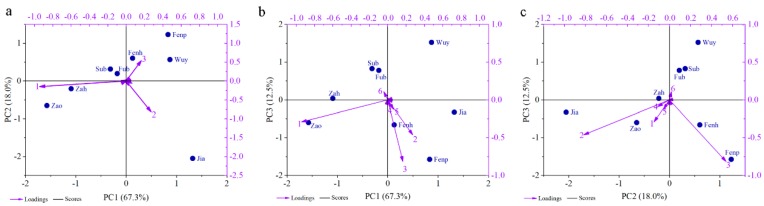
PCA of the emission amount of floral scent compounds separated the eight *P. mume* cultivars. (**a**) 2D type of component plot of PC1 and PC2. (**b**) 2D type of component plot of PC1 and PC3. (**c**) 2D type of component plot of PC2 and PC3. Violet axis represents the loading value of the compounds. Black axis represents the score of the cultivars. Numbers represent the major scent components: 1: benzyl acetate; 2: benzyl alcohol, 3: cinnamyl acetate, 4: eugenol, 5: cinnamyl alcohol, 6: benzyl benzoate.

To categorize the intraspecific cultivars, PCA was performed based on the emission amount of floral volatiles, helping to highlight the similarity and difference in the dataset [5]. As shown in Figure 3, the variances of PC1, PC2, and PC3 were 67.3%, 18.0%, and 12.5%, respectively. The six highest loading values in each PC were selected as main factors, including benzyl acetate, benzyl alcohol, cinnamyl acetate, eugenol, cinnamyl alcohol and benzyl benzoate, which contributed to separate the eight cultivars. In Figure 3a,b, the emission amount of benzyl acetate was a main factor to separate ‘Zao’ and ‘Zah’ from other cultivars. Benzyl alcohol amount was one principal component to distinguish ‘Jia’ from others. In Figure 3a,c, cinnamyl acetate helped to separate ‘Fenp’ and ‘Fenh’ from other cultivars. Benzyl benzoate was the main component to separate ‘Sub’ and ‘Fub’ from others. In Figure 3b,c, ‘Wuy’ was obviously separated from others because of its low emission of VOCs.

### 2.3. Hierarchical Cluster Analysis of Floral Volatiles in the Eight P. mume Cultivars

**Figure 4:**
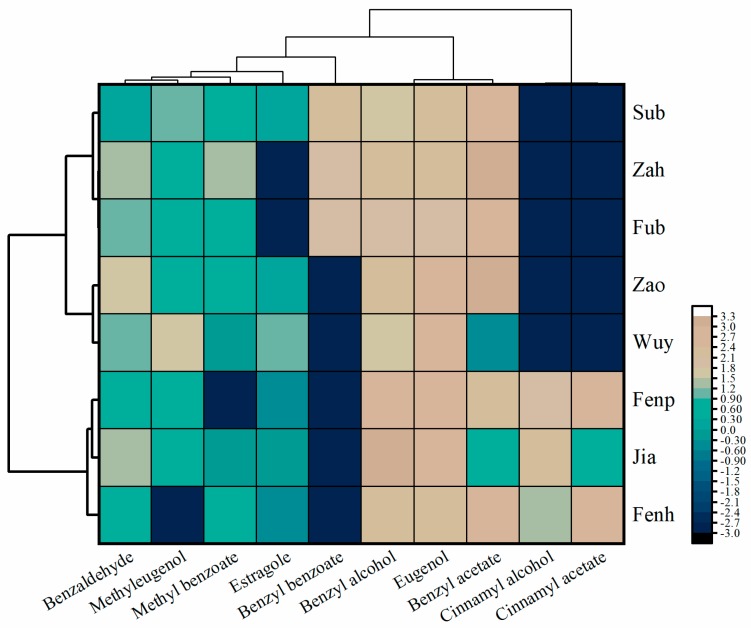
Heatmap of core VOCs and hierarchical cluster analysis of eight *P. mume* cultivars. The colour of the heatmap ranges from dark blue (value, –3.0) to brown (value, 3.3) in the natural logarithmic scale. Data are presented with means of biological replicates. The emission values were normalized by log_10_ transformation.

To explore the relationships among these cultivars, hierarchical cluster analysis was carried out according to the quantities of mainly detected chemicals. As shown in Figure 4, the cultivars clustered together emitting similar representative scent components. The eight cultivars were clustered into two groups. ‘Sub’, ‘Fub’ and ‘Zah’ were grouped into one-subgroup, in which ‘Fub’ and ‘Zah’ were closer, for the near contents of benzyl benzoate and benzyl acetate. ‘Zao’ and ‘Wuy’ were in the other sub-group. ‘Fenp’, ‘Jia’ and ‘Fenh’ were clustered in the other group albeit to the low level of benzyl acetate and cinnamyl acetate in ‘Jia’. ‘Fenp’ and ‘Jia’ were closer as the amounts of cinnamyl alcohol, benzyl alcohol and eugenol in them were significantly greater than in ‘Fenh’.

## 3. Discussion

Floral scents always vary among different ornamental species or cultivars. Both the composition and the amount of the volatiles determine the specific flower fragrance [19]. The number of volatiles emitted from *P. mume* is less than other species, such as bearded iris (219) [10], *Lithophragma* (132) [20], *G. sempervirens* (81) [8], but approximate to *Lilium* [6], *P. mume* hybrids, and *Cymbidium* [21]. Comprehensive analysis of the floral scent profiles indicates that the fragrance may be not straightly connected with the number of compositions but their concentration. The number of components changes slightly from weak-scented ‘Wuy’ to heavy-scented ‘Zao’. Similar phenomenon occurs in *Camellia* that about 30 volatiles were identified in *C. macrosepala* (weak-scented) and *C. buxifolia* (moderate-scented) [4].

The VOCs are also different among intraspecific or interspecific cultivars. In our work, 31 compounds were identified, predominant with aromatic benzenoids and phenylpropanoids, including benzyl alcohol, benzyl acetate, cinnamyl alcohol, cinnamyl acetate and eugenol. Compared to the preliminary study [14], benzyl acetate and eugenol are the major component in several cultivars indeed. Differently, emission amount of benzyl alcohol in the investigated ‘Jia’ is far more than that in other flowers of *P. mume* cultivars. In addition, cinnamyl alcohol and cinnamyl acetate are novel compounds released from *P. mume*. As shown in our analytical data, benzyl acetate, eugenol, benzyl alcohol and cinnamyl acetate contribute to the floral scents of *P. mume*.

Interaction of various VOCs forms the floral aroma that is characterized by a component with a higher odor value (content/olfactory threshold) [22]. Benzyl acetate is an important aromatic component of ripe fruits and perfumes in many ornamental plants, such as Myrtaceae fruit crops [23], *Jasminum sambac* [24], *Zygopetalum maculatum* [25]. Similarly, the floral scents in four of the eight cultivars, including strong-scented ‘Zao’ and ‘Zah’ and moderate-scented ‘Sub’ and ‘Fub’, are dominated by large amounts of benzyl acetate with fruit and jasmine aroma [26]. The cinnamon-fruity aroma of strong-scented ‘Fenp’ is decided by cinnamyl acetate. And floral odor with sweet-flowery-fruity of moderate-scented ‘Fenh’ is characterized by the interaction of benzyl acetate and cinnamyl acetate. Floral aroma of ‘Jia’ is provided by benzyl alcohol and eugenol. The light scented ‘Wuy’ is characterized by eugenol with a spicy clove odor. The high diversity of floral volatiles provides rich germplasm for developing new cultivars with diverse fragrance.

Through comprehensive analysis of the floral volatile compositions in the eight intraspecific cultivars, we conclude that the components of white flowers were similar but different in pink flowers. In white flowers, benzyl acetate and eugenol function as main components. While benzyl acetate, cinnamyl acetate and benzyl alcohol play important roles in the floral aroma of pink flowers. This result inspires us to further investigate about the complex molecular mechanism in our future study.

The synthesis of floral VOCs is mainly regulated by the expression of related genes, enzyme activity and substrate availability involved in the biosynthesis pathways. Our results indicate that benzenoids, especially aromatic esters, are the predominant components contributing to the unique aroma of *P. mume* cultivars. Similarly, the floral fragrance of many plants is dominated by aromatic esters, which are synthesized from the aromatic alcohol and acyl donor catalyzed with the acyltransferase. This has been reported in many studies, for example, acetyl CoA- benzyl alcohol acetyltransferase (*CbBEAT*) from *Clarkia breweri* [27], benzoyl-CoA: benzyl alcohol benzoyl transferase (*PtBEBT*) from *Populus trichocarpa* [28], and alcohol acetyltransferase 1 (*RhAAT1*) from rosa [29]. As several types of alcohol acyltransferases in different species of plants, *CbBEAT* and *RhAAT1* can catalyze a broad range of acyl-CoAs and alcohols to form the relative aromatic esters [12]. Further, the esterification of alcohols, either benzyl or cinnamyl alcohol, results in the synthesis of a number of important fragrance and flavor compounds [26]. This suggests that formation of benzyl acetate and cinnamyl acetate may be catalyzed by one enzyme in *P. mume*, utilizing benzyl alcohol and cinnamyl alcohol as substrates, respectively. Though enzymes, including *PmBEAT* [12] and coniferyl alcohol acetyltransferase (*PmCFAT*) [30], with similar biochemical characteristics in *P. mume* have been reported, the process by which enzymes catalyze cinnamyl alcohol to produce cinnamyl acetate needs further investigation. Comparative transcriptome analysis can be an essential and effective strategy for fully understanding the scent biosynthesis in *P. mume*.

## 4. Materials and Methods 

### 4.1. Plant Material

Eight *P. mume* cultivars with different flower colours grown in Jiufeng international registration park (Beijing, China) were selected, including *P. mume* ‘Fuban Lve’(Fub), *P. mume* ‘Zaohua Lve’(Zah), *P. mume* ‘Subai Taige’(Sub), *P. mume* ‘Zao Yudie’ (Zao), *P. mume* ‘Fenpi Gongfen’(Fenp), *P. mume* ‘Jiangsha Gongfen’ (Jia), *P. mume* ‘Fenhong Zhusha’(Fenh), and *P. mume* ‘Wuyuyu’(Wuy). They bloom during January to February every year. Names of these cultivars are referred to the book: Chinese Mei Flower Cultivars in Color (in chinese press). Flower colours were identified using the Royal Horticultural Society Color Chart (RHSCC).

### 4.2. Floral Scent Collection and Quantitative Determination

The flower scents were collected through dynamic headspace collection method at 12:00–14:00 p.m. Branches with full bloom flowers were clipped in distilled water, and then instantly transported to the laboratory at 25 °C for volatile collection. In each of three experimental replicates, 0.2–0.3 g whole blooming flowers was collected and placed into 100 mL injection vials, which were held for 10 min before using extraction fiber to adsorb the volatiles for 30 min at 30 °C. SPME fiber coated with divinylbenzene/carboxen/polydimenthylsiloxane (50/30 μm DVB/CAR/PDMS) was selected to collect volatiles, as reported in previous studies [31]. The emitted volatiles were analyzed using GC-MS, carried out by Shimadzu QP2010 (Shimadzu, Kyoto, Japan) equipped with a DB-5MS capillary column (30 × 0.25 mm, 0.25 μm thickness, Shimadzu, Kyoto, Japan). The injection temperature was held at 250 °C. Helium was the carrier gas in the split mode with the split ratio at 20 and column flow at 27.0 mL min^−1^. The GC oven temperature started at 40 °C, maintained 2 min, and then increased to 250 °C by 5 °C min^−1^, holding for 6 min. The mass spectrometer interface temperature was 250 °C and the electron potential was set to 0.9 KV with mass scan range of 30 to 300 *m/z* units. The solvent cut time was 3.7 min.

The peak area of every scent compound was integrated to obtain the total ion current, with removing the peaks presented in control sample. Individual compound was tentatively identified by comparing the mass spectra with NIST11 library (the National Institute of Standards and Technology 2011, Shimadzu, Japan). Main compounds (relative amount more than 1%) were confirmed by comparing with authentic standard samples. 20 mg of standard compounds (benzaldehyde, benzyl alcohol, benzyl acetate, estragole, eugenol, methyl benzoate and propionic acid benzyl ester) were diluted using 1 mL of hexane. Then, 20 mg of standard compounds of cinnamyl alcohol, methyleugenol, cinnamyl acetate and benzyl benzoate were diluted using 1 mL of methanol. Further, 50 μL of each diluted solution was then co-added to a total volume of 1ml of hexane to make a mixed solution. Then, 5 μL of this mixed solution was placed into 100 mL capped vial for measurement. In addition, propionic acid benzyl ester as internal standard was added into capped vial in every sample. To improve the repeatability of the internal standard in every sample, 5 μL of propionic acid benzyl ester diluted solution was placed into the same position of the injection vial wall.

### 4.3. Data Analysis

Area normalization was used to calculate the relative content of components. The semi-quantification data was affirmed by standardizing the peak area of each component with the internal standard. PCA was performed with a covariance matrix to calculate the Eigenvalues of principal components. A heatmap of the emission amount of main chemicals and hierarchical cluster analysis of eight *P. mume* cultivars were conducted using Ward’s method, and squared Euclidean distance was chosen as the similarity metric. The cluster analysis and PCA were carried out by OriginPro 2018 (https://www.originlab.com). Microsoft Excel 2016 was used as the statistical tool.

## 5. Conclusions

In total, 31 VOCs were separated and identified from fully bloomed flowers of eight *P. mume* intraspecific cultivars by HS-SPME-GC-MS. Floral scent components varied within intraspecific cultivars. Besides benzyl acetate and eugenol, among the emitted volatiles, benzyl alcohol and cinnamyl acetate also contributed to the floral scent of *P. mume*. The composition of floral aroma from white-flower cultivars were similar, in which benzyl acetate dominated their floral scents. While there were differences in floral volatile compositions from pink flowers. Cinnamyl alcohol and cinnamyl acetate were synthesized only in pink flowers: ‘Fenp’, ‘Jia’ and ‘Fenh’. The high diversity of flower fragrance in *P. mume* provided a germplasm resource for developing new cultivars with a variety of fragrances. Comparative transcriptomic and metabolomic analyses could be an essential and effective strategy for fully understanding scent biosynthesis in *P. mume*.

## Figures and Tables

**Table 1:** Summary of compounds identified in the eight *P. mume* cultivars.

Cultivars	‘Fub’	‘Zah’	‘Sub’	‘Zao’	‘Fenp’	‘Jia’	‘Fenh’	‘Wuy’
RHSCC	NN155B	NN155B	155C	NN155C	62B	63B	68B	60B
Number of compounds	20	20	21	18	23	20	22	19

**Table 2:** Emission amount of main floral scent compounds in the eight *P. mume* cultivars.

Compounds	RI ^1^	Emission Amount [mean ± s.d. (μg·g^−1^ fresh weight·h^−1^)]							
		‘Fub’	‘Zah’	‘Sub’	‘Zao’	‘Fenp’	‘Jia’	‘Fenh’	‘Wuy’
Benzaldehyde	982	10.1 ± 0.7	19.3 ± 14.3	1.5 ± 0.1	34.1 ± 22.0	6.0 ± 3.8	17.6 ± 2.4	6.0 ± 1.6	13.4 ± 3.6
Benzyl alcohol	1036	108.3 ± 21.2	149.2 ± 46.9	38.2 ± 7.6	221.6 ± 112.3	279.3 ± 8.3	1094.0 ± 0.8	227.0 ± 54.2	50.6 ± 9.9
Methyl benzoate	1060	6.3 ± 4.0	24.8 ± 11.3	3.6 ± 2.1	2.2 ± 0.7	tr ^2^	0.97 ± 0.46	6.3 ± 1.3	0.8 ± 0.3
Benzyl acetate	1160	709.1 ± 152.7	1333.1 ± 360.5	778.2 ± 131.4	1691.0 ± 292.8	239.2 ± 1.9	7.10 ± 1.85	620.1 ± 104.6	0.6 ± tr
Estragole	1172	- ^3^	-	1.5 ± 1.5	1.4 ± 0.3	0.4 ± 0.1	1.0 ± tr	0.3 ± tr	9.6 ± 3.2
Cinnamyl alcohol	1243	-	-	-	-	116.1 ± 13.5	138.5 ± 34.1	17.8 ± 5.6	-
Eugenol	1392	88.1 ± 35.6	145.0 ± 85.6	166.6 ± 58.5	406.3 ± 16.3	274.3 ± 4.6	325.9 ± 46.9	168.0 ± 46.1	336.2 ± 54.1
Methyleugenol	1361	3.2 ± 1.0	6.1 ± 2.9	8.4 ± 1.9	5.1 ± 0.9	3.4 ± 0.7	7.2 ± 2.7	tr	32.3 ± 8.9
Cinnamyl acetate	1367	-	-	-	-	810.3 ± 177.1	4.4 ± 1.5	423.5 ± 139.6	-
Benzyl benzoate	1733	95.0 ± 42.4	119.87 ± 38.9	157.5 ± 82.5	-	-	-	-	-

^1^ retention index. ^2^ trace amount, less than 0.1. ^3^ not detected.

## Supplementary Materials

The following are available online at https://www.mdpi.com/1420-3049/25/1/145/s1, Table S1: Relative amounts (>0.1%) of floral scent compounds in the eight *P. mume* cultivars.
