# Supplementary material for: A Comparative Analysis of Floral Scent Compounds in Intraspecific Cultivars of Prunus mume with Different Corolla Colours
Source: Molecules. 2019 Dec 30;25(1):145. doi: 10.3390/molecules25010145 (PMC6982963; doi:10.3390/molecules25010145)
Supplement: Supplementary file 1 [file molecules-25-00145-s001.pdf]

SUPPLEMENTARY INFO

A comparative analysis of floral scent compounds in intraspecific cultivars of *Prunus mume* with different corollas colours

Tengxun Zhang[1#], Fei Bao[1#], Yongjuan Yang[1], Ling Hu[1], Anqi Ding[1], Aiqin Ding[1], Jia Wang[1], Tangren Cheng[1] and Qixiang Zhang[1*]

[1] Beijing Advanced Innovation Center for Tree Breeding by Molecular Design, Beijing Forestry University, Beijing, China; Beijing Key Laboratory of Ornamental Plants Germplasm Innovation & Molecular Breeding; National Engineering Research Center for Floriculture; Beijing Laboratory of Urban and Rural Ecological Environment; Key Laboratory of Genetics and Breeding in Forest Trees and Ornamental Plants of Ministry of Education; School of Landscape Architecture, Beijing Forestry University, Beijing, China

[#]These authors contributed equally to this work.

Correspondence to:

Qixiang Zhang
School of Landscape Architecture, Beijing Forestry University, Beijing 10083, China
e-mail: zqxbjfu@126.com

Supplementary Table

Table S1. Relative amounts (>0.1%) of floral scent compounds in the eight *P. mume* cultivars.

Compounds	RI[1]	Relative amount [mean ± SE (%)]							
		'Fub'	'Zah'	'Sub'	'Zao'	'Fenp'	'Jia'	'Fenh'	'Wuy'
**Phenylpropanoids/Benzenoids**									
Benzaldehyde	982	0.85±0.11	2.00±0.25	0.14±0.02	1.16±0.51	0.72±0.14	1.45±0.25	0.48±0.26	5.83±1.70
4-Methylanisole	983	0.62±0.11	0.56±0.16	0.53±0.05	tr[2]	tr	tr	tr	tr
Benzyl alcohol	1036	7.51±0.05	4.56±0.08	2.53±0.49	7.23±1.87	23.49±0.95	75.43±2.83	13.60±2.57	10.45±3.56
Methyl benzoate	1060	0.70±0.32	1.51±0.27	0.29±0.00	0.09±0.03	tr	0.11±0.05	0.75±0.04	0.47±0.18
Benzyl acetate	1160	83.46±1.10	86.02±2.06	87.26±2.39	84.02±6.24	34.23±2.14	0.84±0.25	68.52±0.55	0.21±0.03
Creosol	1203	-[3]	0.13±0.03	-	-	-	-	0.08±0.03	-
Methyl salicylate	1281	0.12±0.06	-	tr	tr	tr	-	tr	-
Estragole	1172	-	-	0.10±0.04	0.09±0.01	tr	0.13±0.01	tr	7.14±2.05
3-Phenylpropanol	1235	-	-	tr	-	0.09±0.02	0.17±0.04	-	-
Chavicol	1203	-	-	-	-	0.75±0.22	0.21±0.08	-	1.93±0.44
trans-Cinnamaldehyde	1189	-	-	-	-	0.51±0.04	0.40±0.10	-	-
Cinnamyl alcohol	1243	-	-	-	-	6.81±0.32	6.66±1.39	0.88±0.24	-
Eugenol	1392	3.53±0.74	3.08±0.77	7.17±0.02	4.49±1.80	13.63±0.73	13.24±1.40	7.00±1.88	66.59±3.47
Benzenepropyl acetate	1359	-	-	-	-	0.92±0.16	0.08±0.04	0.34±0.11	0.35±0.16
Methyleugenol	1361	tr	0.13±0.03	0.17±0.01	tr	0.09±0.03	0.16±0.06	tr	3.36±0.72
Cinnamyl acetate	1367	-	-	-	-	18.14±2.71	0.08±0.03	7.80±1.07	-
Benzyl benzoate	1733	0.68±0.17	0.71±0.22	1.33±0.08	-	-	-	-	-
**Fatty acid derivatives**									
3-Hexanol	780	tr	tr	tr	-	tr	tr	tr	0.52±0.14
2-Hexanol	780	tr	0.11±0.02	tr	-	tr	0.11±0.06	tr	0.61±0.11
(4E)-4-Hexenyl acetate	992	0.30±0.06	0.31±0.09	0.27±0.07	tr	-	-	0.23±0.06	-
Cyclohexyl acetate	1047	0.63±0.22	0.43±0.10	0.43±0.13	0.17±0.08	tr	-	0.23±0.08	-
2-Hexenyl acetate	992	0.24±0.08	0.81±0.22	0.43±0.07	0.18±0.03	-	-	0.11±0.01	-
Ethylhexanol	995	-	-	0.29±0.02	-	tr	-	-	0.48±0.01
1-Octanol	1059	tr	tr	tr	tr	0.38±0.04	0.58±0.31	-	tr
Nonanal	1104	tr	tr	tr	tr	tr	tr	-	0.58±0.24

Ethyl hexyl acetate	1118	tr	tr	0.24±0.07	tr	-	-	tr	-
Octyl acetate	1183	0.33±0.16	-	0.20±0.04	-	0.09±0.01	-	-	-
**Terpenoids**									
D-Limonene	1018	-	tr	-	tr	-	tr	tr	0.11±0.13
Menthol	1164	-	-	-	-	-	-	-	0.50±0.06
Dihydro-β-ionone	1449	0.08±0.04	0.06±0.02	-	tr	-	-	tr	0.19±0.04
β-Ionone	1457	0.19±0.07	tr	-	tr	0.09±0.06	0.27±0.02	0.07±0.03	tr
